# Population-Level Scale-Up of Cervical Cancer Prevention Services in a Low-Resource Setting: Development, Implementation, and Evaluation of the Cervical Cancer Prevention Program in Zambia

**DOI:** 10.1371/journal.pone.0122169

**Published:** 2015-04-17

**Authors:** Groesbeck P. Parham, Mulindi H. Mwanahamuntu, Sharon Kapambwe, Richard Muwonge, Allen C. Bateman, Meridith Blevins, Carla J. Chibwesha, Krista S. Pfaendler, Victor Mudenda, Aaron L. Shibemba, Samson Chisele, Gracilia Mkumba, Bellington Vwalika, Michael L. Hicks, Sten H. Vermund, Benjamin H. Chi, Jeffrey S. A. Stringer, Rengaswamy Sankaranarayanan, Vikrant V. Sahasrabuddhe

**Affiliations:** 1 Center for Infectious Disease Research in Zambia, Lusaka, Zambia; 2 University of Zambia, Lusaka, Zambia; 3 University of North Carolina at Chapel Hill, Chapel Hill, North Carolina, United States of America; 4 International Agency for Research on Cancer, Lyon, France; 5 Vanderbilt University, Nashville, Tennessee, United States of America; 6 Michigan Cancer Institute, Pontiac, Michigan, United States of America; 7 National Cancer Institute, Bethesda, Maryland, United States of America; 8 University of California, Irvine, Irvine, California, United States of America; Penn State University School of Medicine, UNITED STATES

## Abstract

**Background:**

Very few efforts have been undertaken to scale-up low-cost approaches to cervical cancer prevention in low-resource countries.

**Methods:**

In a public sector cervical cancer prevention program in Zambia, nurses provided visual-inspection with acetic acid (VIA) and cryotherapy in clinics co-housed with HIV/AIDS programs, and referred women with complex lesions for histopathologic evaluation. Low-cost technological adaptations were deployed for improving VIA detection, facilitating expert physician opinion, and ensuring quality assurance. Key process and outcome indicators were derived by analyzing electronic medical records to evaluate program expansion efforts.

**Findings:**

Between 2006-2013, screening services were expanded from 2 to 12 clinics in Lusaka, the most-populous province in Zambia, through which 102,942 women were screened. The majority (71.7%) were in the target age-range of 25–49 years; 28% were HIV-positive. Out of 101,867 with evaluable data, 20,419 (20%) were VIA positive, of whom 11,508 (56.4%) were treated with cryotherapy, and 8,911 (43.6%) were referred for histopathologic evaluation. Most women (87%, 86,301 of 98,961 evaluable) received same-day services (including 5% undergoing same-visit cryotherapy and 82% screening VIA-negative). The proportion of women with cervical intraepithelial neoplasia grade 2 and worse (CIN2+) among those referred for histopathologic evaluation was 44.1% (1,735/3,938 with histopathology results). Detection rates for CIN2+ and invasive cervical cancer were 17 and 7 per 1,000 women screened, respectively. Women with HIV were more likely to screen positive, to be referred for histopathologic evaluation, and to have cervical precancer and cancer than HIV-negative women.

**Interpretation:**

We creatively disrupted the 'no screening' status quo prevailing in Zambia and addressed the heavy burden of cervical disease among previously unscreened women by establishing and scaling-up public-sector screening and treatment services at a population level. Key determinants for successful expansion included leveraging HIV/AIDS program investments, and context-specific information technology applications for quality assurance and filling human resource gaps.

## Introduction

A high incidence of invasive cervical cancer (ICC) is observed in low- and middle-income countries (LMICs) where high quality prevention services are unavailable or inaccessible, awareness about cancer risk is low, healthcare infrastructures are fragmented and dysfunctional, lack of appropriate public health policies and competing priorities detract from making substantive improvements to the status quo [1–4]. While cytology-based screening has been the cornerstone for secondary prevention of cervical cancer in most high-income countries, it remains unavailable or difficult to implement in most resource-constrained settings. Multiple international efforts have been undertaken to develop cost-effective and sustainable alternatives. Screening with visual inspection of the cervix with acetic acid (VIA) has received the most attention globally primarily because of its low cost and facilitation of immediate decisions about treatment (cryotherapy) or referral [5–7]. Observational studies, field demonstration projects and randomized trials have shown that VIA screening can be effective in reducing the incidence and mortality due to ICC [8–10]. Although human papillomavirus (HPV)-based screening is more sensitive and effective than either VIA or cytology [11–13], low-cost and single-visit point-of-care HPV screening assays are not yet widely available, and HPV-positive results invariably need triage by visual screening methods in LMICs [14,15], underscoring the importance of VIA-based cervical cancer screening platforms.

In 2013, the World Health Organization (WHO) endorsed the use of VIA and cryotherapy-based ‘screen-and-treat’ programs in countries without existing cervical cancer services [15]. Yet, several research questions related to implementation of VIA remain, particularly when it is adopted as part of routine health care systems. Are such services scalable at a population-level? Is integration with other vertical disease control programs, such as those for human immunodeficiency virus (HIV)/acquired immunodeficiency syndrome (AIDS) control, practical? Can such screening be implemented in countries with high HIV prevalence? Can operational efficiency be measured through programmatic process and outcome metrics? Can setting-specific challenges in implementation be overcome with locally-developed solutions?

We sought to answer these implementation research questions by conducting an evaluation of the Cervical Cancer Prevention Program in Zambia (CCPPZ), a public-sector program initiated in 2006 that has provided services (as of March 2015) to over 200,000 women using a locally contextualized intervention with simultaneous health system strengthening. This effort represents the largest such national-level initiative and offers a model for scaling-up of cervical cancer prevention services in sub-Saharan Africa and globally.

## Methods

### Setting and Context

Zambia has some of the world’s highest rates of ICC incidence (58.4/100,000 per year) and mortality (36.2/100,000 per year) rates. ICC is the most commonly diagnosed malignancy among the country’s adult population (both sexes combined), and accounts for more than one-third of annual new cancer cases among women. Zambia is also experiencing a generalized HIV epidemic, with HIV sero-prevalence rates of over 14% in the general adult population. The HIV and cervical cancer epidemics intersect significantly, with HIV increasing the risk for cervical cancer and worsening its prognosis. Several national and international efforts, including those funded through the U.S. President’s Emergency Plan for AIDS Relief (PEPFAR), have invested significant resources in upgrading Zambia’s capacity for prevention, care, and treatment of HIV/AIDS over the past decade [16,17].

**Fig 1:**
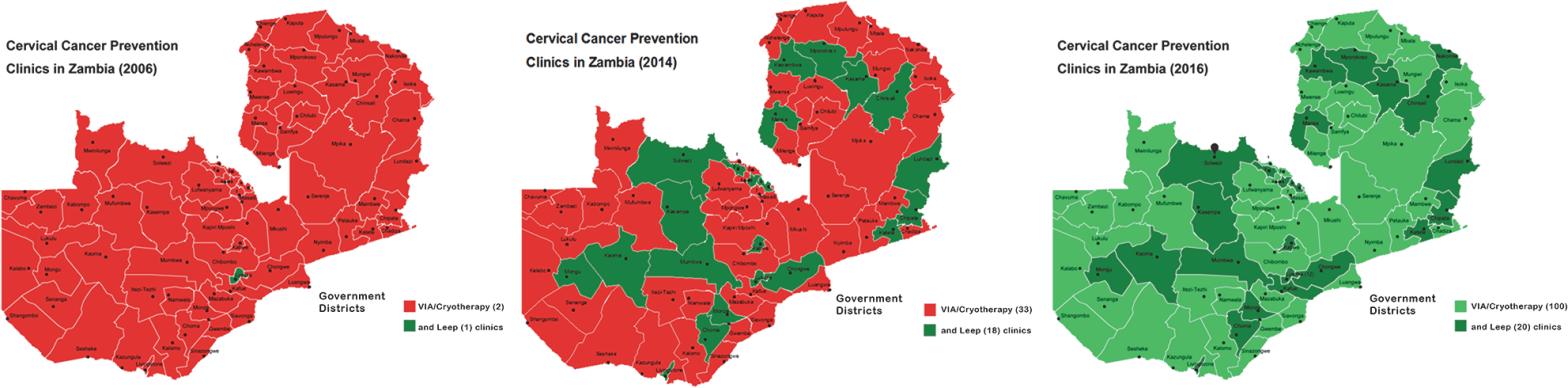
District-level expansion across provinces in Zambia (2006–2014) and projected (in 2016) of the Cervical Cancer Prevention Program in Zambia (CCPPZ).

**Fig 2:**
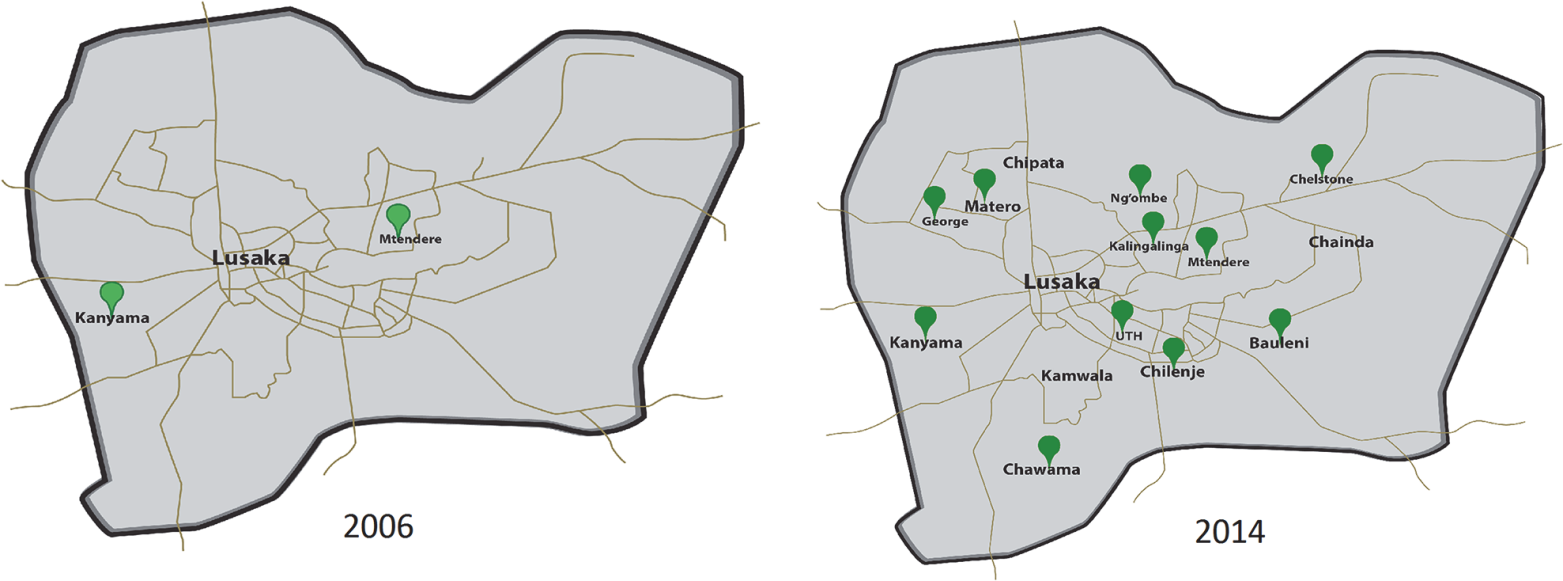
Clinic-level expansion in the Lusaka province (2006–2014) of the Cervical Cancer Prevention Program in Zambia (CCPPZ).

Prompted by pilot research that demonstrated a very high burden of ICC and high-grade cervical precursors among HIV-infected Zambian women newly accessing antiretroviral therapy [18], we began an innovative Zambian-US partnership to initiate CCPPZ in 2006. PEPFAR, through the U.S. Centers for Disease Control and Prevention (CDC), funded CCPPZ as its first-ever cervical cancer prevention initiative focused on HIV-infected women. Local leadership was provided by the Zambian Ministry of Health while program operations were managed by the Center for Infectious Disease Research in Zambia (CIDRZ), a Zambian-US non-profit organization, in collaboration with the Department of Obstetrics and Gynecology of the University of Zambia in Zambia. PEPFAR funding allowed us to initially offer services to the highest-risk HIV-infected women, but the development of the infrastructure and human resources through this funding allowed the program to offer these services to all women in the catchment area, regardless of their HIV status, with low marginal costs. Starting with two public sector clinics in the country’s most populated province (Lusaka) in 2006, CCPPZ now operates in 33 government-run health facilities across all of Zambia’s 10 provinces [19–25]. **(*Fig 1) (Fig 2*).**

### Adaptation of the cervical cancer screening intervention

**Fig 3:**
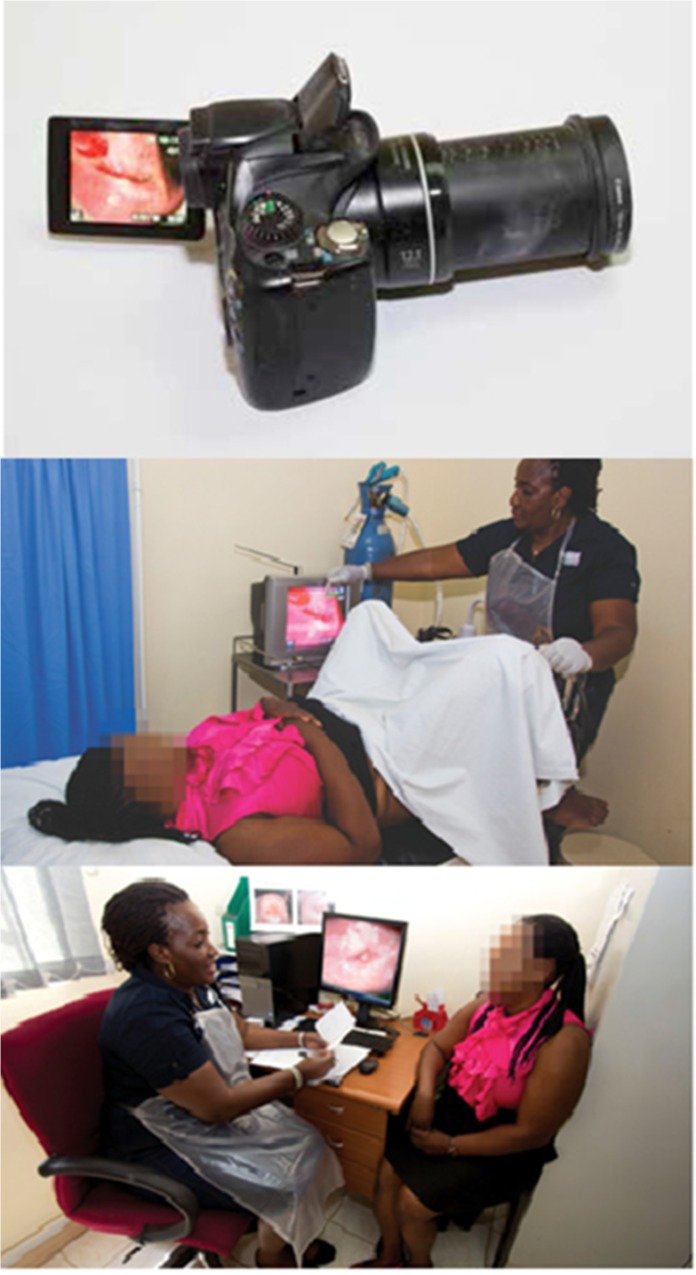
Performing VIA-based screening augmented by digital cervicography in the clinics of the Cervical Cancer Prevention Program in Zambia (CCPPZ). Notes: Nurses in CCPPZ clinics initially perform screening using VIA, after which they use a commercial brand (off-the-shelf) hand-held digital camera *(* ***Upper Panel***) to take photographs of the cervix (cervigrams). Cervigrams are then displayed on a bedside television or camera monitor in real-time (***Middle Panel***), permitting magnification and detailed examination of lesion morphology, including size, margin sharpness, proximity to the transformation zone, degree of extension into the endocervical canal, abnormal vasculature (mosaicism, punctations, atypical blood vessels) and gross characteristics suspicious for ICC. Cervigrams are routinely shown to and discussed with patients during the screening procedure after which they are uploaded by nurses to a clinic computer where they can be (i) electronically transmitted using cellphone network lines to off-site experts (after deidentifcation) for rapid distance consultation (telecervicography), when necessary, (ii) batched and later routinely peer reviewed to form the basis of a rigorous ongoing continuing education and quality assurance program, and (iii) stored with the patient’s electronic medical record. Relevant cervigrams are transmitted by screening nurses to the referral clinic by email, after deidentification, where they are accessible by consultants at the time of patient visits. (***Lower Panel)***.

**Fig 4:**
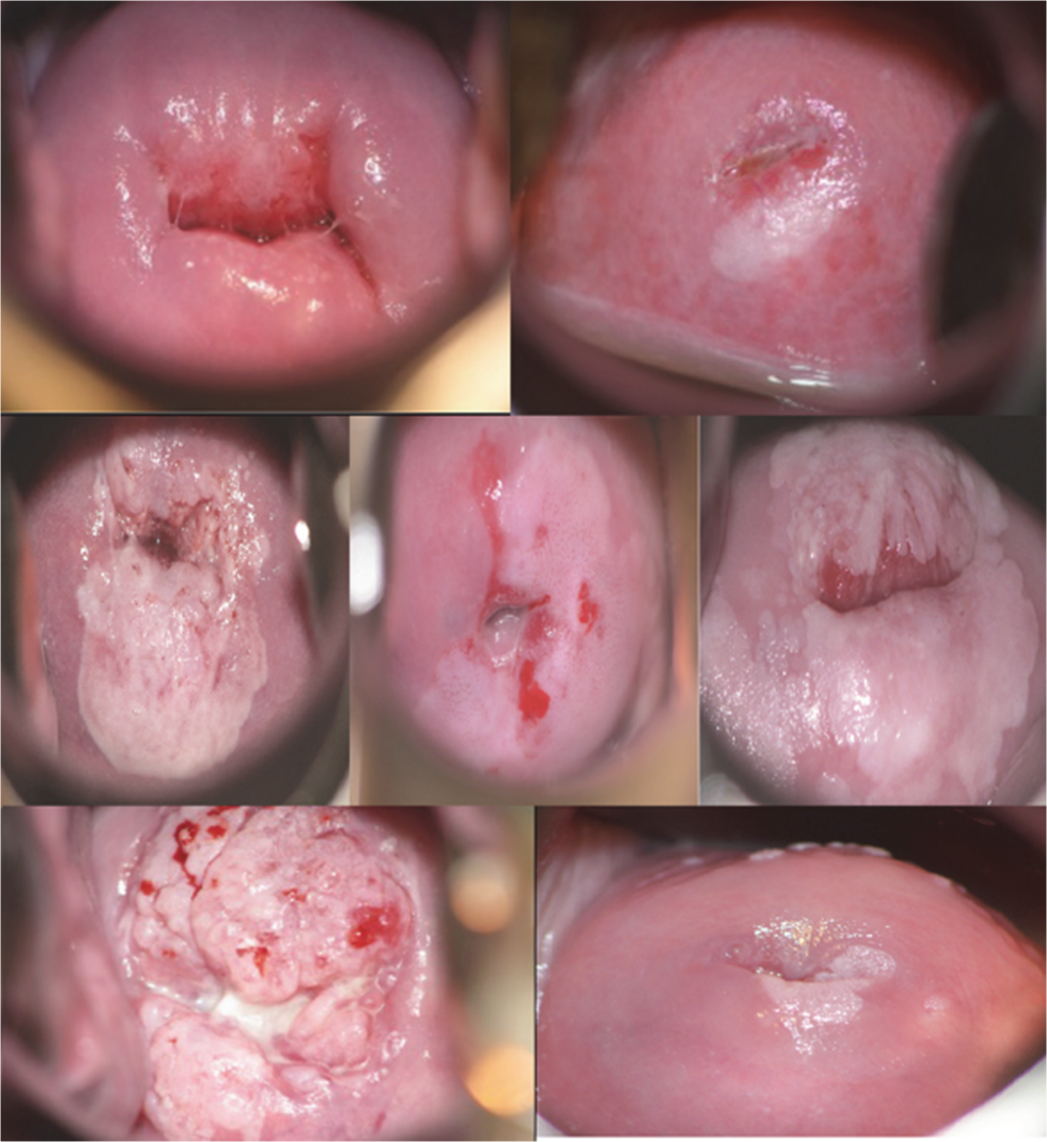
Images of VIA negative, VIA positive-*cryotherapy eligible* and VIA positive-*cryotherapy ineligible* lesions from women undergoing screening in the Cervical Cancer Prevention Program in Zambia (CCPPZ). Notes: Using the following criteria, CCPPZ nurses classify VIA tests results as VIA negative, VIA positive eligible for local ablation with cryotherapy, or VIA positive ineligible for cryotherapy requiring physician evaluation.

Given the concerns about suboptimal sensitivity and lack of an efficient quality assurance mechanism for VIA-based screening, we developed an innovative and locally adapted telecommunications matrix that provided single-visit point-of-care enhanced digital imaging of the cervix (digital cervicography), peer review, quality assurance, continuing medical education, objective record of screening test results, and access to expert opinion through immediate distance consultation, if needed [22]. **(*Fig 3) (Fig 4*)** Treatment decisions were made primarily on the basis of VIA, however, if there were disagreements between VIA and cervicography, the latter was used to make the final decision.

**VIA negative:** Absence of an acetowhite lesion with at least one distinct border (***Top Panel*, *Left***)
**VIA positive, eligible for cryotherapy**: Acetowhite lesion with at least one distinct border, located within or adjacent to the transformation zone, that: occupies <75% or <3 quadrants of the surface of the ectocervix, is completely visualized, can be completely covered by the largest available cryoprobe tip, has no evidence of abnormal vasculature (punctations, mosaicism, atypical blood vessels) and is not suspicious for ICC (***Top panel*, *right***)
**VIA positive, ineligible for cryotherapy**: Acetowhite lesion with at least one distinct border, located within or adjacent to the transformation zone, that has any of the following characteristics:
Occupies ≥3 quadrants or ≥75% of the surface of the ectocervix ***(Middle panel*, *left)***
Has evidence of abnormal vasculature (punctations, mosaicism, atypical blood vessels) ***(Middle panel*, *center)***
Cannot be completely covered by the largest available cryoprobe tip (***Middle panel*, *right***)Is suspicious for invasive cervical cancer (***Bottom panel*, *left***)Extends into the endocervical canal beyond complete visualization (***Bottom panel*, *right***)

### Linking screening to treatment

To address the problem of patient adherence to treatment recommendations we tightly linked screening to treatment in the form of a single-visit ‘screen-and-treat (freeze)’ approach for cryotherapy-eligible lesions, and a two-visit ‘screen-referral-loop electrosurgical excision procedure (LEEP)’ approach (with no intervening punch biopsy except when the lesion is suspicious for ICC) for cryotherapy-ineligible lesions. An acetowhite lesion was considered cryotherapy-eligible if all the following criteria were fulfilled: the lesion involves three-fourths or less of the cervix, does not extend into the endocervical canal, can be fully covered by the cryoprobe, and has no clinical suspicion/evidence of invasive cancer. **(*Fig 4*)** Cryotherapy was provided by trained nurses using a double freeze technique [26] operating independently in public-sector primary care clinics located within the community. Women with complex cryotherapy-ineligible VIA positive lesions requiring LEEP/punch biopsy, as well as women with other gynecologic disorders such as pelvic masses, vulvar lesions, cervical polyps, cervical myomas, abnormal uterine bleeding, etc. were referred for physician evaluation at the University of Zambia’s Gynecologic Cancer Prevention Unit. LEEP or punch biopsy was performed by nurses and physicians in referral clinics, located in facilities equipped with a fully operational surgical theatre in case of hemorrhage. Data were electronically captured at the point-of-care by community health workers and nurses, and in case of HIV-infected women the data were also linked to Zambia’s unique, country-wide electronic medical record system for HIV/AIDS care.

### Adaptations for program expansion

VIA/cryotherapy-based ‘screen and treat’ service platforms were initially expanded into the 12 largest primary public health clinics within Lusaka, the country’s most heavily populated province, with one referral clinic for LEEP and biopsy located at the University of Zambia [21,23]. An identical service platform (VIA/cryotherapy/LEEP) was placed in each of the remaining nine provincial hospitals as well as the large district hospitals within each province. Prior to the establishment of each service platform CCPPZ staff conducted a site visit to meet the Provincial Health Officer, organize the logistics of community awareness and supply chain, and assess the need for administrative, pathology, quality assurance, data collection and communication technology services. All healthcare providers were trained in the CCPPZ’s cervical cancer prevention training program which provides two weeks of didactic and hands-on clinical-mentoring in Lusaka clinics. Every 3 months, nurses from the Lusaka clinics visited facilities in the outlying provinces for purposes of quality assurance and continued medical education. Pathology (LEEP and punch biopsy) specimens were sent by courier to the pathology department at the University of Zambia, the local provincial hospitals, or contracted out to private sector pathologists.

### Program outreach and community mobilization

From the inception of the program, community health workers and community mobilization actions were central components of our health promotion and adherence efforts. Volunteer women with an aptitude for voicing health concerns were selected from communities surrounding their respective screening clinics and trained to conduct cervical health promotion talks to women, to inform them of CCPPZ’s free, walk-in/same-day services using simple, but persuasive, messaging. Women requesting more information or expressing a desire to be screened were ‘navigated’ by these ‘peer educators’ to the CCPPZ clinics.

### Process and outcome metrics

To evaluate the program operations and scale-up of CCPPZ’s services from 2006 to 2013, we analyzed data from the CCPPZ electronic medical records database. We derived key process and outcome metrics that reflected the success of CCPPZ’s prevention services and integration and interaction with and beyond the HIV/AIDS program infrastructure. These metrics included: (i) annual program uptake rate between 2006–2013 and comparison of year-on-year change and differences by age categories and HIV status; these were derived from comparisons of relative proportions and changes in age and HIV-strata over time relative to total women screened, (ii) rates and trends in screening positivity and cryotherapy: comparison of changes over time of the proportion of women screening positive (defined as being screen-positive on either VIA or cervicography, out of the total number of women screened) and those receiving cryotherapy (among total women screened and among women screening positive) and differences by age category groups and HIV status, (iii) proportion of women receiving treatment on the same day of screening (the ‘screen-and-treat’ approach) among screen-positive women that received cryotherapy treatment, (iv) differences in rates of histopathologic detection of cervical precancer (cervical intraepithelial neoplasia grades 2/3 [CIN2/3]) and ICC by HIV status and age categories, and programmatic yield by histopathologic diagnoses, (v) trends in provision of ‘same-day services’ (i.e., proportion of screen-positive women who underwent same-day cryotherapy plus women who had a screening-negative result, out of total women screened) and rates of confirmed CIN2+ (CIN2/3 and ICC) diagnoses among women referred for histopathologic evaluation, (vi) differences in screening positivity rates and rates of treatment of cervical precancer by HIV status.

This outcomes evaluation study of anonymized patient records of a public sector implementation program was deemed exempt from human subjects review or the need for informed consent by the Research Ethics Committee of the University of Zambia.

## Results

### Program uptake and socio-demographics and HIV status of women undergoing screening

A total of 108,330 women were offered screening in 12 public sector clinics in the Lusaka province in Zambia between January 2006 and December 2013, of whom 102,942 (95%) underwent screening. The absolute number of women undergoing screening increased significantly with every calendar year (p-trend<0.001). The socio-demographic and sexual-reproductive characteristics of these women are shown in ***Table 1***. The vast majority (72%) of women attending the screening were between the ages of 25–49 years, i.e., the target age for screening. The relative composition of age-groups of the screened populations remained relatively stable across the screening period of 2006–2013. Almost half (45%) of the women reported their monthly household income being less than 500,000 Zambian Kwacha (approximately equivalent to US Dollars 80/month), and almost two-thirds (67%) had never completed a high-school education. Just under half reported their first sexual intercourse at age 17 or less, and almost three-quarters (74%) reported having multiple (2+) lifetime sexual partners. A large majority (80%) had multiple (2 or more) pregnancies, and almost one-third (30%) had 5 or more pregnancies. A very tiny minority (1.8%) had ever been screened with Pap smears for cervical cancer.

**Table 1:** Socio-demographic and sexual and reproductive characteristics of participants in the Cervical Cancer Prevention Program in Zambia (CCPPZ) during 2006–2013.

Characteristics	Number	Percentage
Women attending screening clinics	108,330	
Women that received screening	102,942	95.0
Age (years)		
<25	19,240	19.8
25–29	20,452	21.0
30–34	19,237	19.8
35–39	14,831	15.2
40–44	9,393	9.6
45–49	5,922	6.1
50+	8,274	8.5
Education		
No formal education	3,998	4.8
Some primary education	14,215	17.0
Primary school completed	14,743	17.6
Some high school completed	23,275	27.8
High school completed	11,343	13.5
College/University	16,199	19.3
Occupation		
Housewife	27,932	35.3
Formal sector	16,969	21.4
Informal sector	23,623	29.8
Others	10,649	13.5
Monthly household income (Kwacha)*		
<50,000	1,550	1.9
50,000–99,999	2,279	2.8
100,000–199,999	8,174	10.1
200,000–499,999	24,968	31.0
500,000+	43,671	54.2
Marital status		
Never married	10,534	15.9
Currently married	42,814	64.8
Separated	1,453	2.2
Divorced	5,141	7.8
Widowed	6,153	9.3
Age at first sexual intercourse		
<15	7,136	7.4
15–17	38,464	39.8
18+	51,162	52.9
Number of lifetime sexual partners		
0–1	25,384	26.3
2–4	57,121	59.1
5+	14,097	14.6
Number of pregnancies		
0–1	17,585	20.0
2–4	43,557	49.5
5+	26,777	30.5
Ever had Pap smear		
No	94,690	98.2
Yes	1,755	1.8

* 1 US dollar = 6,430 Zambian Kwacha (ZMK; using pre-2013 currency base)

**Fig 5:**
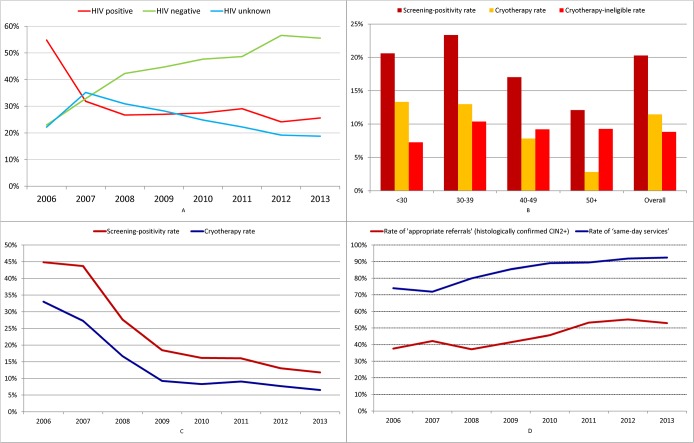
Program process and outcome indicators of the Cervical Cancer Prevention Program in Zambia (CCPPZ). Panel A (Top, left): Trends in HIV status of screened women over the calendar years 2006–2013. Panel B (Bottom, left): Rates of screening positivity, cryotherapy eligibility and cryotherapy-ineligiblity by age categories and overall. Panel C (Bottom, right): Trends in rates of screening positivity and cryotherapy rates over calendar years 2006–2013. Panel D (Top, right): Trends in rates of ‘same day-services’ and rates of ‘appropriate referral’ over calender years 2006–2013.

Overall, just over a quarter of women screened were HIV-positive (28%; 28,529/102,942), about half were HIV-negative (48%; 49,483) and the HIV status was unknown in about a quarter of the women (24%; 24,930). The proportion of HIV-positive women among the total screened population declined from 55% in 2006 to 26% in 2013, while the proportion of women who were HIV-negative increased during the same period from 23% to 56% **(*Fig 5-Panel A*)**. The proportion of women whose HIV status was unknown initially increased from 22% in 2006 to 35% in 2007, but as HIV testing services began being offered in the cervical cancer screening clinics, this proportion steadily fell, and was 19% in 2013.

### Rates of screening-positivity and cryotherapy by age

Among the 102,942 women screened, 1,075 (1%) had uncertain VIA results ***(Table 2)***. Of the remaining 101,867 with conclusive screening results, 20,419 (20%) were screen-positive. The screen positivity rate differed by age and HIV-status and was highest (23%) among women between 30–39 years and lowest (12%) among women >50 years **(*Fig 5-Panel B*)**. The overall screen-positivity rate fell significantly from 45% in 2006 to 12% in 2013 (p-trend<0.001) **(*Fig 5-Panel C*)** and the decline was consistent across HIV-status (data not shown).

**Table 2:** Programmatic process measures (screening and treatment uptake by HIV status) in the Cervical Cancer Prevention Program in Zambia (CCPPZ).

	HIV-positive		HIV-negative		HIV-unknown			Total	
	n (%)		n (%)		n (%)			n (%)	
Women screened	28,529		49,483		24,930		102,942		
Women with uncertain screening results*	368	(1.3)	415	(0.8)	292	(1.2)	1,075		(1.0)
Women with satisfactory screening results	28,161		49,068		24,638		101,867		
Screen-positives**	8,961	(31.8)	6,840	(13.9)	4,618	(18.7)	20,419		(20.0)
Screen-positives treated with cryotherapy at clinics^$^ ^†^	4,289	(47.9)	4,463	(65.2)	2,756	(59.7)	11,508		(56.4)
Screen-positives referred to UTH^$^	4,672	(52.1)	2,377	(34.8)	1,862	(40.3)	8,911		(43.6)
Screen-positives that attended UTH for referral^¥^	2,851	(61.0)	1,238	(52.1)	730	(39.2)	4,819		(54.1)
Screen-positives that received LEEP^$^ ^†^	2,208	(24.6)	798	(11.7)	462	(10.0)	3,468		(17.0)
Screen-positives that received any form of treatment (cryotherapy/LEEP) ^$^ ^†^	6,126	(68.4)	5,080	(74.3)	3,152	(68.3)	14,358		(70.3)
Time interval between screening and cryotherapy									
Screen-positives treated with cryotherapy *(with complete data on screening and treatment dates)*	2,810		2,951		1,766		7,527		
Cryotherapy on same day as screening^§^	1,835	(65.3)	1,903	(64.5)	1,115	(63.1)	4,853		(64.5)
Cryotherapy ≤ one week after screening^§^	349	(12.4)	423	(14.3)	288	(16.3)	1,060		(14.1)
Cryotherapy > one ≤ four weeks after screening^§^	210	(7.5)	197	(6.7)	130	(7.4)	537		(7.1)
Cryotherapy > four weeks after screening^§^	416	(14.8)	428	(14.5)	233	(13.2)	1,077		(14.3)

HIV: HIV: human immunodeficiency virus; UTH: University of Zambia;

* Proportion out of women screened;

** Proportion out of women with satisfactory screening results;

^$^ Proportion out of screen-positives;

^¥^ Proportion out of screen-positives referred to UTH;

^§^ Proportion out of screen-positive women treated with cryotherapy that had complete data on screening and/or treatment dates;

† Some women received both LEEP and cryotherapy; hence these categories are not mutually exclusive.

A total of 11,508 (56%, out of 20,419 VIA screen-positive) women received cryotherapy, and 8,911 (44%) were referred for further evaluation for cryotherapy-ineligible lesions to the referral clinic at the University of Zambia ***(Table 2)***. The rate of cryotherapy (11%, expressed as a proportion of total number of women screened) declined with age (p<0.001) **(*Fig 5-Panel B*)** and with calendar year (31% in 2006 to 6% in 2013, p-trend <0.001) **(*Fig 5-Panel C*)**. The rate of cryotherapy as a proportion of VIA positive women was highest (65%) among women <30 years, and proportionally decreased with age (56% among women 30–39 years, 46% in women 40–49 years, and only 24% in women >50 years) (p-trend <0.001).

Of the 7,527 screen-positive women treated with cryotherapy who had complete information on screening and treatment dates, over two-thirds (65%, 4,853) had treatment on the same day of screening and almost 80% (78.6%, 5,912) within one week of screening ***(Table 2)***. These proportions were similar across different HIV status categories.

### Rates of histopathologically-confirmed cervical precancer and cancer

Among 8,911 screen-positive women referred for physician evaluation, 4,819 (54%) complied with the referral to the University of Zambia (*Table 2*). Among those who complied with referral, histopathology results were available for 3,938 (81.7%) women (denominator for this analysis), of which ICC was present in 710 (18%), CIN2/3 lesions were present in 1,025 (26%), while 2,203 (56%) women had ≤CIN1 lesions. Overall, among women with ICC, 53% were HIV-positive, 27% were HIV-negative and 20% were of unknown-HIV status, while among women with CIN2/3 lesions, the corresponding prevalence of HIV-positive, HIV-negative, and HIV-unknown status was 71%, 18% and 11%, respectively **(*Table 3*)**. Approximately half (50.3%, 357) of the 710 cases of ICC were diagnosed in screen-positive women whose lesions were evaluated with LEEP, as they were cryotherapy-ineligible but not grossly suspicions for invasion, the latter which were diagnosed with punch biopsy ***(Table 3)***.

**Table 3:** Programmatic outcome measures (cervical intraepithelial neoplasia and invasive cancers detected among screen-positives by HIV status) in the Cervical Cancer Prevention Program in Zambia (CCPPZ).

	HIV-Positive		HIV-Negative		HIV-Unknown		Total	
	(n = 28,529)		(n = 49,483)		(n = 24,930)		(n = 102,942)	
CIN and ICC cases detected among women with histopathology results^								
≤CIN 1, n (% of all ≤CIN 1)	1,237	(56.2)	616	(28.0)	350	(15.9)	2,203	(100)
CIN 2/3, n (% of all CIN 2/3)	724	(70.6)	187	(18.2)	114	(11.1)	1,025	(100)
ICC, n (% of all ICC)	376	(53.0)	189	(26.6)	145	(20.4)	710	(100)
CIN2+, n (% of all CIN2+)	1100	(63.4)	376	(21.7)	259	(14.9)	1735	(100)
CIN and ICC cases diagnosed from women undergoing LEEP*								
≤CIN 1, n (% of all ≤CIN 1)	1,005	(57.7)	478	(27.4)	260	(14.9)	1,743	(100)
CIN 2/3, n (% of all CIN 2/3)	658	(72.1)	156	(17.1)	98	(10.7)	912	(100)
ICC, n (% of all ICC)	235	(65.8)	70	(19.6)	52	(14.6)	357	(100)
CIN2+, n (% of all CIN2+)	893	(70.4)	226	(17.8)	150	(11.8)	1,269	(100)
No. of CIN and ICC cases detected per 1000 women screened^								
≤CIN 1, n (95%CI)	43.4	(41.1–45.8)	12	(11.5–13.5)	14	(12.7–15.6)	21	(20.5–22.3)
CIN 2/3, n (95%CI)	25.4	(23.6–27.3)	3.8	(3.3–4.4)	4.6	(3.8–5.5)	10	(9.4–10.6)
ICC, n (95%CI)	13.4	(12.1–14.8)	3.8	(3.3–4.4)	5.8	(4.9–6.8)	6.9	(6.5–7.5)
CIN2+, n (95%CI)	38.6	(36.4–40.9)	7.6	(6.9–8.4)	10.4	(9.2–11.7)	16.9	(16.1–17.7)
No. of women screened to detect one case of CIN and ICC^								
≤CIN 1, n (95%CI)	23.1	(16.1–33.0)	80.3	(61.4–104.5)	71.2	(48.7–103.1)	46.7	(38.7–56.4)
CIN 2/3, n (95%CI)	39.4	(27.5–56.2)	264.6	(206.6–332.1)	218.7	(152.7–303)	100.4	(83.5–120.4)
ICC, n (95%CI)	75.9	(53.2–107.1)	261.8	(204.3–328.8)	171.9	(119.2–241.6)	145	(121–172.8)
CIN2+, n (95%CI)	25.9	(18.1–37.1)	131.6	(101.1–169.5)	96.3	(66.0–138.3)	59.3	(51.7–77.0)

HIV: human immunodeficiency virus; CIN: cervical intraepithelial neoplasia; LEEP: loop electrosurgical excision procedure, ICC: invasive cervical cancer;

^Histopathology results were available among 3,938 women,

* Histopathology results were available from 3,012 LEEP specimens

Over the eight-year period (2006–2013), among every 1,000 women who underwent screening, 10 (95%CI: 9.4–10.6) were detected with histologically-confirmed CIN2/3 lesions, and 6.9 (95%CI: 6.5–7.5) were detected with histologically-confirmed ICC **(*Table 3*)**. In other words, the nurse-led ‘screen-referral-LEEP’ approach led to the detection of one case of CIN2/3 for every 100 women screened, one case of ICC for every 145 (95%CI: 121–172.8) women screened, or one case of CIN2+ (precancer or cancer) among every 59.3 (95%CI: 51.7–77.0) women screened **(*Table 3*)**. Annualizing the cumulative detection rates into an incidence rate, the histologically-proven detection alone would approximately equate to an annual rate of 117/100,000 women (albeit with substantial differences in rates by HIV status ***[Table 3]***), which is more than twice than the present IARC/WHO-reported ICC rate of 58.4/100,000 in Zambia.

### Trends in rates of provision of ‘same-day services’ and histopathologically-confirmed cervical precancer and cancer

Overall, among the 98,961 (102,942 screened minus 3,981 screen-positives treated with cryotherapy who had missing information on screening and/or treatment dates) women with complete screening data, the program provided ‘same-day services’ to a total of 86,301 (87%) women, which included 4,853 (5%) screen-positives who underwent same-visit cryotherapy and 81,448 (82%) who were VIA-negative and were counselled about their risk and advised follow-up after 3–5 years (if HIV-negative) and annual follow-up (if HIV-positive). The rate of ‘same day services’ increased between 2006 and 2013 from 88% to 94% (p<0.001) (***Fig 5-Panel D***). In effect, only one in ten women undergoing screening is required to undertake a second visit (to a referral facility). Among women who underwent histopathologic evaluation, the overall rate of CIN2+ lesions was 44.1% (1,735/3,938). This rate improved significantly from 41% in 2006 to 48% in 2013 (p-trend<0.001) (***Fig 5-Panel D***).

### Differences in screening positivity, treatment and referral rates, and histologically-confirmed CIN2+ rates by HIV status

Women with HIV were twice as likely to be detected as VIA-positive than HIV-negative women (31.8% vs. 13.9%, crude relative risk [RR]: 2.9, 95% CI = 2.8–3.0, p<0.001). Among VIA screen-positive women, as compared to HIV-negative women, women with HIV were less likely to be treated by cryotherapy (48% vs. 65%, crude RR: 0.73, 95% 0.71–0.75, p<0.001), more likely to be referred to UTH for histologic evaluation of cryotherapy-ineligible lesions (52% vs. 34%, crude RR: 1.5, 95% 1.4–1.6, P<0.001), and more likely to have histologically-confirmed CIN2+ (i.e., CIN2/3 and ICC) lesions (47.1% vs. 37.9%, crude RR: 1.5, 95% CI = 1.3–1.7, p<0.001).

## Discussion

The results of this programmatic evaluation demonstrate that VIA-based screening services can be scaled-up at a population-level when adopted as part of the routine health care system in a low-resource, high disease burden country. Cervical cancer prevention services can benefit by efficient linkages with HIV/AIDS care and treatment services and, with time, such services can be offered to all women at need, regardless of HIV status. Our evaluation shows that the operational efficiency of the program as measured by rates of ‘same-day services’ and histopathologically-confirmed precancer and cancer improves with experience and sustained efforts in quality improvement. Setting-specific challenges in implementation can be overcome with locally-developed solutions, particularly with sufficient political will and stakeholder support. The findings emanating from this implementation research study could provide stimulus for replicating such national-level program expansions in sub-Saharan Africa, as well as in other resource-constrained settings globally. Although the cervical screening program in Zambia heavily relied on the HIV care infrastructure for implementation and scaling-up, the broader lesson for settings with a lesser HIV burden is that cervical screening services can be successfully developed and scaled-up in routine public health services by innovative integration with other vertical health initiatives.

Our study has highlighted the substantially high burden of histologically-confirmed precancerous and cancerous (CIN2+) lesions that is uncovered by the establishment of a population-based screening program. It exemplifies the critical need for investments in oncologic surgery and radiation therapy alongside screening platforms, in order to avoid unnecessary deaths, suffering, and social consequences of detected but untreated disease. It also underscores the importance of continued global efforts to expand HPV vaccination.

A retrospective critique and evaluation suggests six distinguishing facets of this program that contributed to the success of the scale-up of the implementation.

First, the leveraging of the momentum and resources of an ongoing, funded vertical health initiative (HIV care and treatment) allowed the development of capacity for the prevention of a non-communicable disease (cervical cancer). Focusing on the provision of services for high-risk HIV-infected women initially allowed a firm rooting of our program and provided a foundation for sustainability, but did not hamper or preclude eventual wider expansion of services to the general (HIV-uninfected) women in the community, as evidenced by the increasing proportion of HIV-negative women over the program expansion period **(*Fig 5-Panel A*)**. Using existing infrastructures for implementing screening can only help in the long-run in terms of costs, expertise and sustainability.

Second, we adapted the screening intervention to local circumstances instead of rigorously retrofitting approaches that are successful, but in radically different environments. While our approach of screening promoted task shifting (doctors to nurses), we harnessed the power of mobile technology to address human resource gaps by allowing distance consultation to improve clinical decision making. Embedding the basic principle of cervical cancer prevention with visual-based screening in a public health context and linking the initial encounter within a matrix of an affordable and locally available telecommunications technology (‘electronic cervical cancer control—eC3’) allowed us to roll-out standardized, quality-assured, cervical cancer screening and real-time distance consultation at remote sites across Zambia where it would never have been possible before [22].

Third, we heavily invested in a surgical excision (LEEP) infrastructure and expanded diagnostic services to facilitate management of complex cervical lesions that exceeded the therapeutic limitations of cryotherapy. The diagnosis and treatment of complex cervical lesions not amenable to local ablation by cryotherapy is as critical as, if not more than, providing same-visit screen-and-treat services for cryotherapy-eligible lesions. This was particularly critical for HIV-positive women who were more likely to require referral for histologic evaluation than HIV-negative women. Screening without linkages to treatment is unethical, hence both ‘single-visit screen-and-treat’ and ‘screen-referral-LEEP’ were frontline components of our comprehensive cervical cancer prevention program. The surgical excision component was a part of the initial programmatic vision itself and not an after-thought. This ensured that treatment of precancerous lesions was feasible for both low- and high-grade precancerous lesions, as reflected by the fact 70% of screen-positive women who were eligible for treatment received it. It also provided the basis for evaluating the most critical histologic outcomes (i.e., CIN2+) for monitoring programmatic impact. LEEP also served the dual purpose of diagnosis and treatment for the vast majority of women diagnosed with CIN3 or more severe lesions such as preclinical, occult invasive cancer.

Fourth, we constantly assessed all phases of the program through a rigorous process of monitoring and evaluation of interim outcomes. Our use of routine programmatic indicators (e.g., screening uptake rates by age and HIV status, rates of cryotherapy-ineligible lesions, screening positivity and CIN2+ detection rates by age and HIV status, rates of ‘same day services’ and ‘appropriate referrals’) provided ongoing evidence on the role of improvements in the quality of screening and treatment services over time [19,25]. For instance, the VIA positivity rate dropped from 45% to less than 15% over an 8-year period indicating the learning curve of the providers following supervision and field monitoring [19]. Similar impact of the learning curve of the test providers and field supervision on VIA positivity rates over time has been demonstrated in other studies as well [7–11,27,28]. These process and outcomes indicators were among the only clinical effectiveness metrics in the absence of a well-functioning population-based cancer registry and/or a widely-used national-level citizen identification system to measure declines in cancer incidence over time.

Fifth, we strengthened the existing healthcare delivery system as we gathered evidence and assessed needs, on-the-go, as an innovative approach to sustainability and long-term success. Whether to immediately implement cervical cancer prevention services with meagre resources, or working first to develop well-functioning health systems, was no longer an issue for debate. We arrived at innovative solutions **(*Table 4*)** to overcome critical programmatic challenges posed by the less-than-optimal existing public health infrastructure, all while providing the expansion of service provision activities. These approaches to problem-solving provide some of the very few, if any, ‘hands-on’ examples for implementing similar programs in resource constrained settings. They reflect the product of critical thinking by a close knit team of pragmatic clinicians (doctors and nurses) and public health program staff, consisting of Zambian nationals and an expatriate U.S. board-certified gynecologic oncologist (GPP) living within the country, and represent vital and intermediate steps in the development and sustenance of efficient healthcare delivery systems in such settings.

**Table 4:** Critical Problems and Practical Local Solutions in the Cervical Cancer Prevention Program in Zambia (CCPPZ) *(list not exhaustive*, *for illustrative purposes only).*

*Problem(s)*	*Practical solution*	*Impact*
Weak opportunistic cytology-based cervical cancer screening infrastructure in the pubic system; no source of independent funding for cervical cancer prevention	Horizontal integration of a cervical cancer prevention intervention into a pre-existing HIV care and treatment infrastructure; VIA as the main approach	Facilitated population-level implementation and scale-up of intervention
High cost of colposcopy; no quality assurance for VIA	Introduction of innovation: “electronic cervical cancer control”—eC3	Reduction in over and under treatment
Shortage of gynecologists to provide expert opinion	Web consultation: Immediate access to physician opinion through telemedicine	Access to clinical expert for decision making
Low level of community awareness; myths and misconceptions about cervical cancer	Used peer educators and traditional social, political and medical infrastructures for health promotion	Increased patient uptake
Paper records not managed well	Point-of-care electronic data collection	More efficient and secure data system
Pathology bottleneck (delays in results reporting due to unavailability of pathology services)	Liaison with UTH pathology department; “one off” supplementation of supplies; use of private sector pathology services	Efficiency of pathology services significantly improved
Chemoradiation unavailability/ referral challenges	Formal Organizational Linkages with the National Cancer Centre in Lusaka	More efficient referrals
Bottleneck of early stage cervical cancer cases requiring radical surgery	Established gynecologic oncology surgical service in public and private sector	Slight relief of bottleneck
Complex acetowhite lesions ineligible for cryotherapy	Developed capacity for loop electrosurgical excision procedure (LEEP)	Ability to evaluate and treat cryotherapy-ineligible lesions
Deficit of cervical cancer prevention clinicians	Local training program for nurses and doctors	Increased supply of clinical personnel
Reluctance of women undergoing screening, with unknown HIV status, to be tested in nearby HIV care and treatment clinics	Implemented HIV counseling and testing within “screen and treat” clinics, by screening nurses	Marked increase in HIV testing rates
Management of microinvasive cancers in young women desirous of fertility potential	Developed algorithm for conservative management of women with microinvasive cancer	Avoidance of unnecessary loss of fertility
Unreliable and insecure procurement, storage and distribution of equipment and supplies	Hiring of Procurement Officer and procurement system	Efficient and secure procurement system
Lack of local ownership	Persistent mentoring of Zambians at all levels of leadership; Integration of “screen and treat” service platforms in government-operated clinics; Limiting role of CIDRZ to “technical assistance”	Increased ownership by Ministry of Health, Ministry of Community Development, Mother and Child Health
Increased demand for services, particularly in rural areas	Implementation of outreach program in the form of village-based screening camps; Training of a cadre of trainers, i.e., TOTs	Rapid expansion of services into rural areas
High cost and unreliable supply of compressed gas leading to frequent interruptions of services	Use of alternative local ablation strategies, i.e., cold coagulation and Cryopen	Uninterrupted “screen and treat services
Lack of available local leadership to champion the cause of cervical cancer prevention	CIDRZ supported full-time, on-the-ground support for U.S. gynecologic oncologist	Program initiated
Inefficient management	Creation of management infrastructure and stratification of organization into separate units: administrative, health promotions, clinical, information communication technology (ICT) and quality improvement.	Improved efficiency
Limited funding for programmatic innovations	Participation in international student intern and fellowship programs, e.g., Fogarty, Global Health Corp	Support for innovative developments
Evaluation of programmatic outcomes	Development of an all-electronic database of screening results, key sociodemographic and sexual and reproductive history covariates; linked to histologically-confirmed results	Improved assessment of programmatic impact

Sixth, women detected with invasive cervical cancer are treated with radical surgery or megavoltage radiotherapy/chemoradiotherapy at the national cancer center in Lusaka (Cancer Diseases Hospital). This matrix type of cancer center has been organized by the Zambian government at the University of Zambia Teaching Hospital in close proximity to and interaction with pathology, diagnostic imaging and other tertiary care services in the teaching hospital that permits comprehensive cancer diagnosis and treatment services for cancer patients within the country.

In collaboration with the Zambian government (Ministry of Health, Ministry of Community Development, Mother and Child Health, and the University of Zambia), CCPPZ presently (September 2014) supports 33 ‘screen and treat’ clinics and 18 LEEP clinics located throughout the country’s 10 provinces **(*Fig 1) (Fig 2*)**. According to national cervical cancer control plans there will be 100 ‘screen and treat’ clinics and 20 LEEP clinics by December 2016. Nevertheless, the present model of establishing ‘screen and treat’ clinics in government-operated stationary public health facilities has only made incremental gains in terms of women screened per annum. A recent analysis of our programmatic data revealed that using our present model, assuming an increase in patient uptake of 10% per annum, it would require 17 additional years to reach the target population of 1.5 million Zambian women.

We have thus embarked upon a phase of screening which is intended to facilitate a radical and exponential uptake of services. The Zambian government has (as of March 2015) officially adopted CCPPZ as its national cervical cancer prevention program and it will be administered out of the Ministry of Health. Building on our present clinic infrastructure and telecommunications matrix it will focus on the provision of services in an outreach context and will include screening camps and village-based screening in collaboration with Ministry of Chiefs and Traditional Affairs, with local chiefs and chieftainesses, traditional marriage counsellors and traditional healers as primary advocates and facilitators. This new approach continues our use of the philosophy of adaptive innovation which, in principle, incorporates respect for local beliefs and culture while integrating new ideas and technology that fit the local circumstances.

## References

[1] Editorial.The right to cervical cancer services in southern Africa. Lancet 2012; 380: 1622 10.1016/S0140-6736(12)61931-X 23141600

[2] Murillo R, Almonte M, Pereira A, Ferrer E, Gamboa OA, Jeronimo J et al Cervical cancer screening programs in Latin America and the Caribbean. Vaccine 2008; 26 Suppl 11: L37–L48. 10.1016/j.vaccine.2008.06.013 18945401

[3] Forman D, Bray F, Brewster DH, Gombe Mbakawa C, Kohler B, Piñeros M, et al Cancer Incidence in Five Continents, Vol. X (electronic version): Lyon: IARC Available: http://ci5.iarc.fr. Accessed 2014 August 15.

[4] Ferlay J, Soerjomataram I, Ervik M, Dikshit R, Eser S, Mathers C, et al GLOBOCAN 2012 v1.0, Cancer Incidence and Mortality Worldwide: IARC CancerBase No. 11 [Internet]. Lyon: IARC Available: http://globocan.iarc.fr. Accessed 2014 August 15.

[5] Sankaranarayanan R, Nessa A, Esmy PO, Dangou JM. Visual inspection methods for cervical cancer prevention. Best Pract Res Clin Obstet Gynaecol 2012; 26: 221–232. 10.1016/j.bpobgyn.2011.08.003 22075441

[6] Szarewski A. Cervical screening by visual inspection with acetic acid. Lancet 2007; 370: 365–366. 17678998 10.1016/S0140-6736(07)61171-4

[7] University of Zimbabwe/JHPIEGO Cervical Cancer Project.Visual inspection with acetic acid for cervical-cancer screening: test qualities in a primary-care setting. University of Zimbabwe/JHPIEGO Cervical Cancer Project. Lancet 1999; 353: 869–873. 10093978

[8] Gaffikin L, Blumenthal PD, Emerson M, Limpaphayom K. Safety, acceptability, and feasibility of a single-visit approach to cervical-cancer prevention in rural Thailand: a demonstration project. Lancet 2003; 361: 814–820. 12642047 10.1016/s0140-6736(03)12707-9

[9] Sankaranarayanan R, Esmy PO, Rajkumar R, Muwonge R, Swaminathan R, Shanthakumari S, et al Effect of visual screening on cervical cancer incidence and mortality in Tamil Nadu, India: a cluster-randomised trial. Lancet 2007; 370: 398–406. 17679017 10.1016/S0140-6736(07)61195-7

[10] Shastri SS, Mittra I, Mishra GA, Gupta S, Dikshit R, Singh S, et al Effect of VIA screening by primary health workers: randomized controlled study in Mumbai, India. J Natl Cancer Inst 2014; 106: dju009 10.1093/jnci/dju009 24563518 PMC3982783

[11] Sankaranarayanan R, Nene BM, Shastri SS, Jayant K, Muwonge R, Budukh AM, et al HPV screening for cervical cancer in rural India. N Engl J Med 2009; 360: 1385–1394. 10.1056/NEJMoa0808516 19339719

[12] Arbyn M, Ronco G, Anttila A, Meijer CJ, Poljak M, Ogilvie G, et al Evidence regarding human papillomavirus testing in secondary prevention of cervical cancer. Vaccine 2012; 30 Suppl 5: F88–F99. 10.1016/j.vaccine.2012.06.095 23199969

[13] Ronco G, Dillner J, Elfstrom KM, Tunesi S, Snijders PJ, Arbyn M, et al Efficacy of HPV-based screening for prevention of invasive cervical cancer: follow-up of four European randomised controlled trials. Lancet 2014; 383: 524–532. 10.1016/S0140-6736(13)62218-7 24192252

[14] Sahasrabuddhe VV, Parham GP, Mwanahamuntu MH, Vermund SH. Cervical cancer prevention in low- and middle-income countries: feasible, affordable, essential. Cancer Prev Res (Phila) 2012; 5: 11–17. 22158053 10.1158/1940-6207.CAPR-11-0540 PMC3586242

[15] WHO WHO guidelines for screening and treatment of precancerous lesions for cervical cancer prevention 2013; Geneva: WHO. 24716265

[16] Ullrich A, Ott JJ, Vitoria M, Martin-Moreno JM, Atun R. Long-term care of AIDS and non-communicable diseases. Lancet 2011; 377: 639–640. 10.1016/S0140-6736(11)60233-X 21334535

[17] Bendavid E, Holmes CB, Bhattacharya J, Miller G. HIV development assistance and adult mortality in Africa. JAMA 2012; 307: 2060–2067. 10.1001/jama.2012.2001 22665105 PMC3434229

[18] Parham GP, Sahasrabuddhe VV, Mwanahamuntu MH, Shepherd BE, Hicks ML, Stringer EM et al Prevalence and predictors of squamous intraepithelial lesions of the cervix in HIV-infected women in Lusaka, Zambia. Gynecol Oncol 2006; 103: 1017–1022. 16875716 10.1016/j.ygyno.2006.06.015 PMC2748907

[19] Mwanahamuntu MH, Sahasrabuddhe VV, Blevins M, Kapambwe S, Shepherd BE, Chibwesha C, et al Utilization of cervical cancer screening services and trends in screening positivity rates in a 'screen-and-treat' program integrated with HIV/AIDS care in Zambia. PLoS One 2013; 8: e74607 10.1371/journal.pone.0074607 24058599 PMC3776830

[20] Mwanahamuntu MH, Sahasrabuddhe VV, Kapambwe S, Pfaendler KS, Chibwesha C, Mkumba G, et al Advancing cervical cancer prevention initiatives in resource-constrained settings: insights from the Cervical Cancer Prevention Program in Zambia. PLoS Med 2011; 8: e1001032 10.1371/journal.pmed.1001032 21610859 PMC3096609

[21] Mwanahamuntu MH, Sahasrabuddhe VV, Pfaendler KS, Mudenda V, Hicks ML, Vermund SH, et al Implementation of 'see-and-treat' cervical cancer prevention services linked to HIV care in Zambia. AIDS 2009; 23: N1–N5. 10.1097/QAD.0b013e3283236e11 19279439 PMC2747794

[22] Parham GP, Mwanahamuntu MH, Pfaendler KS, Sahasrabuddhe VV, Myung D, Mkumba G, et al eC3—a modern telecommunications matrix for cervical cancer prevention in Zambia. J Low Genit Tract Dis 2010; 14: 167–173. 10.1097/LGT.0b013e3181cd6d5e 20592550 PMC3809081

[23] Pfaendler KS, Mwanahamuntu MH, Sahasrabuddhe VV, Mudenda V, Stringer JS, Parham GP. Management of cryotherapy-ineligible women in a "screen-and-treat" cervical cancer prevention program targeting HIV-infected women in Zambia: lessons from the field. Gynecol Oncol 2008; 110: 402–407. 10.1016/j.ygyno.2008.04.031 18556050 PMC2745977

[24] Chirwa S, Mwanahamuntu M, Kapambwe S, Mkumba G, Stringer J, Sahasrabuddhe V, et al Myths and misconceptions about cervical cancer among Zambian women: rapid assessment by peer educators. Glob Health Promot 2010; 17: 47–50. 10.1177/1757975910363938 20595342 PMC4123628

[25] Mwanahamuntu MH, Sahasrabuddhe VV, Blevins M, Kapambwe S, Shepherd BE, Chibwesha C, et al Monitoring the performance of "screen-and-treat" cervical cancer prevention programs. Int J Gynaecol Obstet 2014; 126: 88–89. 10.1016/j.ijgo.2014.01.006 24810795 PMC4123629

[26] Sellors JW, Sankaranarayanan R. Colposcopy and treatment of cervical intraepithelial neoplasia A beginner's manual. 2003; Lyon: IARC.

[27] Ngoma T, Muwonge R, Mwaiselage J, Kawegere J, Bukori P, Sankaranarayanan R. Evaluation of cervical visual inspection screening in Dar es Salaam, Tanzania. Int J Gynaecol Obstet 2010; 109: 100–104. 10.1016/j.ijgo.2009.11.025 20152973

[28] Muwonge R, Manuel MG, Filipe AP, Dumas JB, Frank MR, Sankaranarayanan R. Visual screening for early detection of cervical neoplasia in Angola. Int J Gynaecol Obstet 2010; 111: 68–72. 10.1016/j.ijgo.2010.04.024 20570259

